# Welfare Assessment: Correspondence Analysis of Welfare Score and Hematological and Biochemical Profiles of Dairy Cows in Sardinia, Italy

**DOI:** 10.3390/ani11030854

**Published:** 2021-03-17

**Authors:** Federica Loi, Giovannantonio Pilo, Giulia Franzoni, Rossana Re, Francesca Fusi, Luigi Bertocchi, Ugo Santucci, Valentina Lorenzi, Sandro Rolesu, Paola Nicolussi

**Affiliations:** 1 Osservatorio Epidemiologico Veterinario Regionale della Sardegna, Istituto Zooprofilattico Sperimentale della Sardegna “G. Pegreffi”, Via XX Settembre 9, 09125 Cagliari, Italy; federica.loi@izs-sardegna.it (F.L.); sandro.rolesu@izs-sardegna.it (S.R.); 2 Istituto Zooprofilattico Sperimentale della Sardegna “G. Pegreffi”, Via Duca degli Abruzzi 8, 07100 Sassari, Italy; gantonio.pilo@izs-sardegna.it (G.P.); rossana.re@izs-sardegna.it (R.R.); paola.nicolussi@izs-sardegna.it (P.N.); 3 Italian National Reference Centre for Animal Welfare, Istituto Zooprofilattico Sperimentale della Lombardia e dell’Emilia Romagna “Bruno Ubertini”, Via A. Bianchi 9, 25124 Brescia, Italy; francesca.fusi@izsler.it (F.F.); luigi.bertocchi@izsler.it (L.B.); valentina.lorenzi@izsler.it (V.L.); 4 Italian Ministry of Health, Directorate-General for Animal Health and Veterinary Medicines, Viale Giorgio Ribotta 5, 00144 Roma, Italy; u.santucci@sanita.it

**Keywords:** farm animal welfare, biosecurity, dairy cow, correlation matrix, laboratory parameters

## Abstract

**Simple Summary:**

Defining animal welfare definition is still controversial, and a definition is difficult to establish. Furthermore, welfare detection is often complicated and subject to different interpretations. This work aimed to provide valid indicators to evaluate the welfare of dairy cows. The Animal Welfare and Biosecurity Evaluation form (AWB-EF) checklist developed and validated by the Italian National Centre of Reference for Animal Welfare (CReNBA) was submitted to 16 Sardinian dairy cattle farms. Blood samples from the 230 Holstein breed dairy cattle housed in these farms were analyzed for hematological parameters. Correlation analysis revealed a strong association between AWB-EF (considered as gold standard) and laboratory parameters, indicating correspondence between the health and welfare status of the animals. Our study clearly indicates that the use of a validated checklist in combination with the identification of well-known laboratory parameters can be a fundamental tool for veterinarians to detect stress conditions early.

**Abstract:**

The need for animal welfare definition and assessment is increasing worldwide, and several studies have been conducted to help fill the knowledge gaps regarding the welfare of cattle. However, further studies are needed to provide valid synthetized measures for welfare evaluation. The aim of this study was to assess the welfare status of 16 Sardinian dairy cattle farms, based on the developed Animal Welfare and Biosecurity Evaluation checklist (AWB-EF) and the corresponding hematological, biochemical, and electrophoretic profiles of these animals. Considering the AWB-EF as gold standard, blood samples were collected from 230 Holstein breed dairy cattle, aged between 3 and 8 years, out of the periparturient period, and with no clinical signs of specific pathologies. Principal Component (PC) and correlation analyses were performed to simplify phenomena interpretation and assess positive/negative associations. Four PCs were able to explain 76% of the total variability, and six laboratory parameters were strongly associated with the AWB-EF score (Spearman’s correlation coefficient ≥ 0.40, *p*-Value < 0.05), reflecting the real health status of the animals. Given the complexity of animal welfare as a multidimensional concept and the need to include both animal-based and non-based measures in welfare evaluation, the present work represents a sound basis for future evaluation and veterinary health planning.

## 1. Introduction

The original concept of animal welfare, developed over 50 years ago by Brambell (1965) [1], was updated by Lawrence and Stott [2], who defined animal welfare as an ethical concern for the mental and physical health of animals over which we have a degree of control, in 2010. However, defining and measuring animal welfare remains controversial [3]. As underlined by Devitt et al. in 2018, if, on the one hand, there is a need to take the relationship between farmers and animals into account when considering farm animal welfare standards, then on the other hand, there is limited understanding of how the nature of this relationship influences welfare outcomes, and thus welfare assessment would require a multidisciplinary approach [4].

The need for animal welfare has been underlined by the World Organization for Animal Health (OIE), who recommended that “veterinarians should be the leading advocates for the welfare of all animals, recognizing the key contribution that animals make to human society through food production, companionship, biomedical research, and education” [5]. Increased attention has been given to farm animal welfare in developed countries, especially given the expansion of intensive animal production systems that improve profit and efficiency but challenge the conscience of many consumers [6].

Furthermore, the need for measurable outputs relevant to animal health and welfare, and that are able to determine whether the welfare program is effective, efficient, and transparent, is increasing worldwide [7].

Poor environmental conditions can affect several homeostatic functions and reduce the productive and reproductive performances of livestock. Stress factors and poor welfare can also compromise the host immune system and lead to increased susceptibility to diseases among animals [8]. Farm animal welfare should be viewed as a global condition, where the effects of infectious and non-infectious stressors cannot be easily discriminated and can overlap, challenging the host’s immune system [9]. In fact, the innate immune system can rapidly respond to both infectious and non-infectious stressors, such as metabolic stress conditions, psychological stress, high/low temperatures, oxidative stress, and hypoxia [8].

To fill the knowledge gaps regarding cattle welfare, several studies have attempted to develop scientifically valid methods for assessing welfare [10,11,12,13,14,15,16,17,18,19,20,21,22]. Some of these studies primarily focused on improving external factors, management-based measures, or non-animal-based measures (N-ABMs), which affect welfare without considering the reactions and consequences for the animals [17,18]. Indeed, most of the methods used have not been refined or validated, possibly because they aimed to detect illness rather than welfare [19,20]. Thus, resource-based assessment cannot answer questions about animal welfare. For all these reasons, attempts have been made worldwide to develop animal-based measures (ABMs) to estimate the actual welfare of animals [21,22]. Conversely, the European Food Safety Association (EFSA) has provided evidence that in some cases N-ABMs may be more efficient than ABMs; thus, it follows that both ABMs and N-ABMs are necessary to obtain a holistic approach and achieve an effective overall classification of animal welfare at the farm level [23,24,25]. 

Currently, about two million dairy cows are reared in the Italian national territory, with more than half of these in the north of the country [26]. Despite this large number of animals, there is no official protocol for evaluating the welfare of dairy cows that uses ABMs, N-ABMs, and risk assessment. In 2017, the Italian National Centre of Reference for Animal Welfare (CReNBA) developed a simple and easy-to-use on-farm protocol for assessing the welfare of dairy cows in loose housing systems. They then carried out expert opinion elicitation to characterize a list of management and housing factors potentially associated with negative or positive welfare outcomes in dairy cows [25].

Welfare assessment requires a multidisciplinary approach, and the CReNBA Animal Welfare and Biosecurity Evaluation form (AWB-EF) monitor both ABMs and N-ABMs in dairy cattle farms. Health is a key component of welfare and health status (e.g., presence/absence of disease, organ function, metabolic processes, and internal body condition) is primarily monitored using hematological and biochemical tests [27,28,29]. 

In this study, we aimed to assess whether the level of welfare estimated by the AWB-EF positively correlated with laboratory parameters (metabolic profile, electrophoresis, and blood count), which are regarded as indicators of health status. To achieve this goal, an in-depth investigation on livestock welfare and biosecurity status was conducted in a broad sample of Sardinian dairy cattle herds using the CReNBA AWB-EF as gold standard, and its correlation with laboratory parameters was assessed. Considering that there is no specific official indication about how laboratory parameters relate specifically to animal welfare (except for hemoglobin levels in calves, Italian Health Ministry D.L. 331, 1st September 1998) [30], this study could represent an important starting point for identifying simple and easily detectable parameters that can help monitor animal welfare.

## 2. Materials and Methods 

### 2.1. Study Context 

Sardinia is an Italian island in the center of the Mediterranean Sea (40°03′ N 9°05′ E) with a total surface area of 24,100 km^2^ and a population density of 69 inh/km^2^ [31]. Administratively, the Sardinian region comprises five provinces, as established by the Regional Law of 4 February 2016: Nuoro, Sassari, Oristano, South of Sardinia, and Cagliari (metropolitan city), with 377 municipalities in total [32]. Given the low population density and the unpolluted environment, the Sardinian economy is mostly based on agropastoral activities [33]. Across the whole island, around 9,200 dairy cow farms are regularly recorded in the Italian Veterinary National database, with a dairy cow population of about 260,000 animals. Sardinian cattle breeding is mainly intended for meat production (85%; 7700 farms). The main area dedicated to beef production is located in the north east of the region, (Sassari and Nuoro provinces), while most of the bovine livestock for milk production is located in the center of the island (Oristano province), where the territory mainly comprises large lowland areas [26]. 

### 2.2. The Animal Welfare and Biosecurity Evaluation Form 

For the purpose of this work, the AWB-EF checklist was implemented by two trained veterinarians, during the farms’ official controls, in 16 dairy cow farms in Sardinia (Italy) between 2013–2015. The checklist was provided by the CReNBA, aimed to monitor the welfare conditions of dairy cows, and consisted of 90 items divided into 5 sections (A1, A2, A3, B, C) (Table S1). In detail, section (A) is divided into (A1) farm management (22 items, i.e., number of stockpeople and their training, animal grouping, inspection, type of handling, feeding strategy, water provision, cleanliness, bedding material and calving pen management), (A2) structures and equipment (29 items, i.e., features about space availability, calving pen, bedding material, floor, feeding space, water points, facilities for sick animals, milking machine, temperature, humidity, ventilation, gas concentration, and artificial lighting), (A3) animal-based measures (18 items, i.e., details about lameness, mortality rate, body condition score, treatments for mastitis, integument alterations, and mutilations; cleanliness of flank, leg, and udder).The other sections are (B) biosecurity (15 items, i.e., general measures against rodents, insect, precautions for entry of strangers, disinfection, quarantine, carcasses management, animal movement management, and disease prevention) and (C) risks and alarm system (5 items, i.e., noise, fire and ventilation alarm, and electricity generator). Each item contributed equally to generating a percentage score for each section, constituting five different outcomes. Furthermore, general information on the number of animal breeds, as well as the animals’ average age, breed, the number of lactating cows and milk production (kg of milk by animal per day), was collected by the AWB-EF checklist for each farm. Animals in the periparturient period were excluded to avoid confounding bias in laboratory parameters detection [34]. The AWB-EF was provided in paper format, and all data were subsequently collected and stored in an ad hoc password-protected electronic database using a closed response data collection instrument (Microsoft Excel, Microsoft Corporation, Redmond, WA, USA).

### 2.3. Laboratory Analysis

Laboratory parameters that were able to verify whether the farm conformed to Italian (D.Lgs.n.146/2001; D.Lgs.n.126/2011) and European legislation (Directive 98/58/EC; Directive 2008/119/EC) were chosen based on EFSA reports [23,24,35,36,37,38,39,40,41], to evaluate whether the AWB score corresponded with the cows’ welfare conditions on the basis of the five outcomes. During the veterinarian’s official visits, blood sampling was performed on Holstein cows between 3 and 8 years old at different stages of lactation. Individual blood samples were collected from the animals’ coccygeal vein before feeding. They were transferred into vacuum tubes containing EDTA anticoagulant for hematological profiling and then into serum gel separator tubes without anticoagulant for both biochemical profiling and analysis of the electrophoretic pattern of serum proteins. The samples were placed in a container with ice and forwarded to the laboratory within 2 h. The serum was separated by laboratory centrifugation at 3500× *g* for 10 min at 4 °C, placed in 1.5-mL tubes, and stored at −20 °C until analysis, while the EDTA tubes were analyzed within 3 h. Hematological determinations were made using an ADVIA 2120 automatic hematology analyzer (Bayer Healthcare, Siemens, Monaco, Germany) with software that allows blood determination in cattle. Biochemical parameters were analyzed using a Dimension RXL chemistry analyzer (Siemens), and serum protein electrophoresis was carried out using an INTERLAB G26 Automated Agarose Gel Electrophoresis Analyzer (Interlab, Rome, Italy). The set of parameters used for biochemical profiling are provided in Table S2. 

### 2.4. Sample Size and Inclusion/Exclusion Criteria

Dairy cow livestock breeding (not mixed species farming system) business’s start and end dates were available, and business activities for the entire study period (i.e., business start date before on or before 1 January 2013 and business end date after on or after 31 December 2015) and the available animal census data were essential farm characteristics to be included in this study. If the farmer had more than one farm with a unique fiscal code, or the farms were inactive for the entire study period, or the farm had missing business start data and/or end data or animal census data for one or more of the study years, the farm was excluded. 

The minimum number of animals to be observed is defined by the Welfare and Biosecurity Manual for dairy cows, performed by CReNBA and specific for the AWB-EF checklist (Table S2). Otherwise, given that programmed statistical analysis (factorial analysis) is strongly influenced by the ratio of sample size (N) to the number of variables being analyzed (*p*) [42], a specific sample size calculation was carried out before proceedings, following MacCallum et al., 1999 [43]. Given the large number of variables collected (31) and the number of hypothesized factors (5), a total sample size of 200 animals (plus 15% of these animals to account for drop out) was considered appropriate. A total of 16 randomly selected dairy cow farms were included in the study. Animals raised on the 16 farms were included in the study, except for animals in the periparturient period and those outside of the age range (3–8 years). 230 samples from 230 randomly selected cows were collected and analyzed.

For each farm, the following baseline characteristics were collected: location (latitude and longitude), province, municipality, opening date, number of animals on farm, age, race, number of lactating cows, milk production, details of previous disease (i.e., skin lesions, lameness, mastitis), mortality rate (overall mortality year rate), and number of animals with laboratory parameters within the normal range. 

### 2.5. Statistical Analysis

Data quality and completeness were tested based on an extensive data check. Descriptive analyses based on mean (SD), median (I–III quartile) and frequency (percentages) were performed to evaluate the distribution of the parameters at baseline compared with the laboratory reference values. A correlation matrix was performed to evaluate association between variables. The linear or nonlinear nature of the relationship between the dependent variable (i.e., percentage score for each section) and each of the continuous independent variables was assessed graphically. When a linear relationship was assumed, its strength was initially evaluated through bivariate analyses by means of the Spearman non-parametric correlation coefficient. When relationships were assumed to be other than linear, mathematical transformations (i.e., log-normal) were applied. The homogeneity of the samples between farms was graphically evaluated to exclude bias generated by the within group correlation or sub-group populations.

The main hypothesis to be tested by the statistical analyses was the correlation between the percentage score for each section of the AWB-EF checklist with the laboratory results (electrophoresis, metabolic profile, and blood count; Table S3). A principal component analysis (PCA) with a varimax rotation was used to make the factors orthogonal and more interpretable, and to extract the laboratory patterns and confirm the number of dimensions underlying the set of variables [44]. The number of retained components was determined according to eigenvalues (≥2.0), scree plot examination and interpretability [45].

Given the non-normal distribution of all the variables included, to evaluate the strength of the relationship (association) between the AWB-EF scores of each checklist section and the laboratory parameters, the nonparametric Spearman’s correlation coefficient was estimated. A correlation matrix and correlation graphs were produced. Any outlying observation that appeared to deviate markedly from other observations in the sample was checked. Only 8 values significantly deviated from the mean, and 3 of these were related to reporting errors (and consequently corrected). The other five were included, considering that, in such cases, outliers may be due to random variation or may indicate something scientifically interesting [46]. A high statistically significant correlation (coeff. ≥ 0.7, *p*-Value < 0.05) means that two variables have a strong relationship, while a weak correlation means that the variables are hardly related. All statistical tests were two-sided, and a *p*-Value less than 0.05 was considered statistically significant. Statistical analyses were performed using Stata13 (StataCorp, Stata statistical software, Release 13; StataCorp LP, College Station, TX, USA) and R-software (Version 3.6.2; R-Foundation for Statistical Computing, Vienna, Austria).

## 3. Results

### 3.1. Descriptive and Principal Component Analysis Results

The baseline characteristics of each farm and thirty-four laboratory parameters were collected from 230 dairy cows raised on 16 extensive farms. The farm baseline characteristics are reported in Table 1, while the laboratory parameters and comparisons with the reference ranges are reported in Table 2. Farms included in this study were well-distributed around the Sardinian island. Totally, 5110 Holstein cows were bred on these 16 farms, with an average of 216 animals (SD = 131) and a median age of 4 years (I–III quartile = 3–9), as established by the inclusion criteria. On each farm, an average of 109 (SD = 63) animals were in the lactating period, with an associated average milk production of 31 (SD = 2.44) kilograms of milk per cow per day. About 15% of the animals on each farm reported skin lesions, 6% lameness, and 30% has undergone treatment for mastitis. The overall mortality rate was about the 2% of the overall population. In total, 217 out of 230 cows (94%) showed laboratory parameters within the normal range.

**Figure 1:**
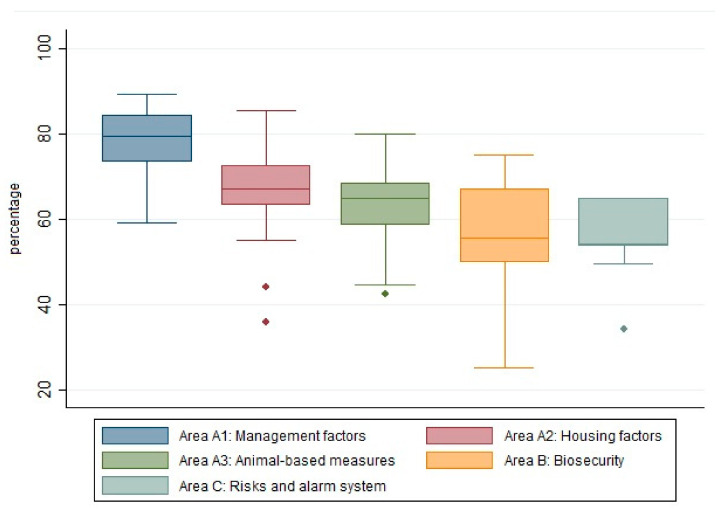
Baseline distribution of the five sections of the AWB-EF: management factors (blue boxplot), housing factors (red boxplot), animal-based measures (green boxplot), biosecurity (yellow boxplot), and risk and alarm system (grey boxplot). Through the box plot, it is possible to observe the median value (horizontal line within the box), quartiles, variability (length of the box) and outliers (dots) of the scores obtained for each section.

The five AWB-EF sections were evaluated and summarized in Figure 1. The average value of section A1 (farm management) was 76.9% (SD = 8.8), while section A2 (structures and equipment) showed an average percentage of 64.5% (SD = 12.3) and section A3 (animal-based measures) had a lower average value of 62.9% (SD = 10.1). Finally, sections B (biosecurity) and C (risks and alarm system) of the AWB-EF checklist showed lower averages, with values of 52.3% (SD = 11.3) and 54% (SD = 11.3), respectively. 

**Figure 2:**
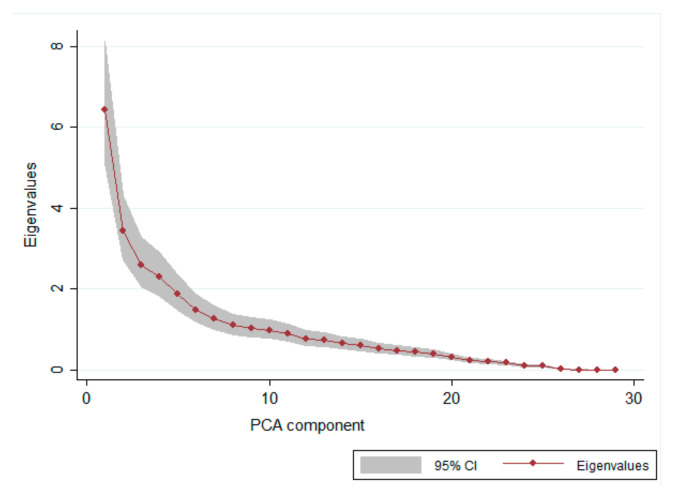
The scree plot, used to determinate the appropriate number of principal components, shows the eigenvalues against the PCA component number.

Based on PCA analysis, according to the eigenvalues and the scree plot (Figure 2), four components are able to explain why 76% of the total variance was maintained.

The first component (fact1) was able to explain 43% of the total variance and was positively characterized by alkaline phosphatase (ALP), glutamic-pyruvic transaminase (GPT), white blood cells (WBC), red blood cells (RBC), hemoglobin (HGB), hematocrit (HCT) and the total number of lymphocytes (LYMPH) and basophils (BAS). The second component (fact2) explained 18% of the total variance and was positively characterized by α1-globulin (A1GB), α2-globulin (A2GB), β-globulin (BGB), γ-globulin (GGB), WBC count, platelets (PLT), average platelet volume (MPV), and the total number of monocytes (MONO) and basophils. The third PCA component (fact3) was positively characterized by WBC, RBC and HGB count, average corpuscular volume (MCV), average hemoglobin content (MCH), medium corpuscular hemoglobin concentration (MCHC), MPV, and the total number of neutrophils (NEUT) and eosinophils (EOS), explaining 9% of the total variance. The last component (fact4) explains 6% of the total variance and was positively characterized by albumin (ALB), BGB, gamma-glutamyl transpeptidase (GGT), GPT, HGB, HCT, and MCV.

Considering the main parameters that characterized each PCA factor, the four dimensions highlighted by the PCA were named as “Overall Welfare”, “Electrophoresis”, “Blood count” and “Metabolic profile”, respectively, rather than dimension 1, 2, 3 and 4 as conventionally used [44].

### 3.2. Results of Correlation Analysis

Five correlation matrices were computed to evaluate the association between A1, A2, A3, B, and C scores with the laboratory parameters of Pearson’s correlation coefficient and correspondent *p*-Values (Table 3).

The correlation matrix was interpreted by considering the positive relationships between variables as ‘very strong’ when the Spearman’s correlation coefficient was higher than 0.70, ‘strong’ when it was between 0.40–0.69, ‘moderate’ when it was between 0.30–0.39, ‘weak’ when it was between 0.20–0.29, ‘negligible’ when it was < 0.20, and likewise for the negative relationships between negative correlation values [47]. In particular, 16 parameters were statistically correlated with the score A1–management factor, and the Spearman’s correlation coefficient of 0.54 and −0.48 indicates a strong positive relationship with amplitude of hemoglobin distribution (HDW) and strong negative relationship with GPT (Table 3, Score A1), respectively. All 12 variables statistically correlated with the score A2–housing factors and showed a weak or negligible positive or negative coefficient, except for GPT and LYMPH, which showed a negative and positive moderate correlation, respectively (Table 3, Score A2), as well as neutrophil-lymphocyte ratio (NRL). A strong negative correlation was described by a 0.52 coefficient between the score A3–animal-based measures and A1GB, while the A2GB was moderately positively correlated with the score (coefficient = 0.37) (Table 3, Score A3), and the other five variables showed moderate (BGB, MCHC, BAS) or weak correlation (GGB, MPV). Between the 10 variables statistically correlated with score B–biosecurity, a very strong negative relationship was identified with A1GB (Table 3, Score B), a very strong positive relationship with A2GB (Table 3, Score B), and a strong negative relationship with GPT, with Spearman’s correlation coefficients of −0.71, 0.72 and −0.51, respectively. The score C–risk and alarm system were statistically significantly correlated with seven variables, but only blood urea nitrogen (BUN) and HDW showed strong positive relationships (coefficients = 0.66 and 0.40, respectively) (Table 3, Score C). 

## 4. Discussion

The present study evaluated the welfare status of 230 dairy cows on 16 dairy cattle farms in Sardinia, testing the association between the welfare score detected by the AWB-EF checklist and the individual hematological and biochemical parameters observed. Considering the welfare protocol score as gold standard, and by correlating it with laboratory results, we were able to estimate whether the data recorded by the protocol reflected the health status of the animals. The five different AWB-EF sections reflecting the laboratory patterns individuated with PCA analysis, and each score showed strong and statistically significant association with one or more of the laboratory parameters. 

Considering the PCA results, welfare status in farm management could be well described by the electrophoresis, blood count and metabolic profile. Besides, the correlation matrix results described a strong association between AWB-EF checklist scores and laboratory parameters such as A1GB, A2GB, GPT, HDW, LYMPH and BUN. 

Serum protein levels, including α1-globulin and α2-globulin, are correlated with important functions of organic synthesis (mainly in the liver), and their levels depict the animals’ capacity to cope with growth and production demands [48]. Electrophoresis has been used to monitor the ability of young bulls to adapt to a different environment, and researchers observed statistically significant variations in levels of either albumin, α-globulin, β-globulin or γ-globulin several times after arrival at the new farming center [49]. Another study described abnormality in electrophoretic parameters in calves after transportation stress, and particularly α-globulin peak was the most predictive of diseases in Holstein Friesian cattle among the clinical immunological parameters evaluated [50]. In the same study, hematological parameters were monitored in animals after transportation stress, and researchers observed that WBC was the most predictive of disease among the hematological parameters evaluated. Nevertheless, the differences between leucocyte subsets were not monitored in that study [50]. Overall, WBC and the variation of different leukocyte populations can be monitored to evaluate the sanitary status of the herds [51]. In our study, a strong association of AWB-EF was also observed with two biochemical parameters: GPT and BUN. Evaluation of the serum activities of hepatic enzymes, including GPT, is routinely used to monitor liver health status, whereas BUN levels are inversely correlated with the decline of kidney function [51,52,53,54]. Correct functionality of these two vital organs is strongly correlated with animal welfare. 

The health status of animals is one criterion of welfare assessment, and it is important to monitor management and housing factors, which are strongly related to a lower incidence of disease and mortality, mental comfort, absence of stress, good appetite, body homeostasis, and maintenance of proper animal welfare levels [55]. Assessing welfare requires detailed knowledge of the available scientific information, since the definition involves describing how well the animals experience their environment based on the best possible judgement of their situation. Such information is necessary to avoid errors in interpreting a given measure and cannot be based solely on science or on data collected from experiments or laboratory analysis [56]. Proper management conditions are essential for organisms to function normally [57], and hematological and biochemical tests may help veterinarians to understand animals’ welfare status based on factors other than the presence/absence of disease [27,58,59].

Several studies used hematological and biochemical parameters to assess animal health and welfare status, both in livestock and small animals [16,22,25,48,52,60,61,62,63,64]. Nevertheless, the use of laboratory parameters requires the collection of samples on the farm and, depending on the parameters used, can entail quite high costs in the purchase of reagents and require time for sample collection and analysis. Thus, welfare protocols are a more immediate and less expensive method for judging the living conditions of animals. Few reports have focused on welfare scores in cows, and more in-depth studies are needed to detect the causes of possible deviations from normal reference ranges. Animal-based measures alone are not enough to ensure a complete evaluation of animal welfare [41]. As a result, applying welfare protocols requires a more general farm evaluation carried out by well-trained veterinarians, which could be difficult to organize. The combination of both welfare protocol and laboratory parameters could be a valid tool to assess a robust estimation of both animal health and welfare status.

In this study, the score assigned by the welfare protocol mainly corresponded with the animals’ health status, and the analysis showed that some laboratory patterns may be particularly useful indicators of welfare. Otherwise, the results of correlation analysis must be carefully considered, as their extrapolation could be dangerous. Correlation analysis only considers the linear relationship between two variables (i.e., other variables that could influence the response variable are not studied) and could be affected by outliers. Furthermore, correlation analysis does not establish if one variable is dependent and the other is independent. Therefore, correlation analysis provides information about the strength and the direction (positive or negative) of a relationship between two continuous variables, but a strong correlation does not imply a cause-and-effect relationship. 

Furthermore, a limit of this study is that information (i.e., day in milk, amount of milk production, age, lactating period) is collected overall by farm and is not available for each animal. Thus, considering that the single animal was the epidemiological unit for the correlation analysis, this information was not included in the final analysis, generating a possible bias considering that these conditions could significantly influence blood and milk parameters [65].

We can conclude that the average values of the hematological and biochemical parameters fell within the range of reference values. Mainly strong or moderate associations have been highlighted between the average welfare score of the dairy farms and laboratory analysis, suggesting that the welfare protocol score mainly reflected the real health status of dairy cattle. The use of a validated checklist in combination with the identification of few well-defined parameters, able to synthetize the health and welfare status of the animals, can be a fundamental tool for veterinarians to detect stress conditions early. Although more in-depth analysis is needed to provide not only an association between measures and the checklist but to quantify and model this association, the results obtained in this study are a strong starting point for future research. Finally, it is necessary to underline that health is a key component of welfare, and welfare assessment requires a multidisciplinary approach, so a laboratory evaluation of the health status of animals cannot be considered the sole criteria for determining animal welfare.

## Figures and Tables

**Table 1:** Baseline characteristics of the 16 dairy cow farms included in the study.

Features	Estimates
Location	
Lat	39.24621–40.89128
Long	8.435341–9.76654
Activities date	2013–2015
No. of animals breed	216 (131)
Age of the animals	4 (3–9)
Breed	230 (100%) Holstein cows
No. of lactating cows	109 (63)
Milk production (kg of milk/animal/day)	31 (2.44)
Skin lesions	
>30% of the animals	0 (0%)
15–30% of the animals	1 (6%)
<15% of the animals	(15%)
Lameness	
>8% of the animals	4 (25%)
4–8% of the animals	5 (31%)
<4% of the animals	7 (44%)
Mastitis	
>80% of the animals	0 (0%)
40–80% of the animals	2 (12%)
<40% of the animals	14 (88%)
Overall mortality year rate	2 (1–5)
No. of animals with normal range parameters	217 (94%)

Estimates are reported as mean (SD); median (I–III quartile); frequency (%); percentage (minimum-max) collected by farm.

**Table 2:** The diagnostic biochemical, electrophoretic, and hematological parameters of the 230 dairy cows tested, indicated as an abbreviation (unit of measurement) and expressed mean (standard deviation) or median (I–III quartile), based on their normal or not-normal distribution and laboratory reference ranges.

Parameters	Abbreviation (unit of measure)	Estimates	Reference Range
ALFA1 globulins	A1GB (%)	3.1 (2.7)	3.0–4.5
ALFA2 globulins	A2GB (%)	6.3 (5.2–9.6)	4.4–8.5
Albumin	ALB (g/dL)	2.7 (0.22)	1.8–3.2
Alkaline phosphatase	ALP (U/L)	63 (48–90)	50–300
BETA globulins	BGB (%)	15.3 (3.02)	9–15.8
Total Bilirubin	TB (mg/dL)	0.12 (0.01)	0.05–0.5
Creatinine	CREA (mg/dL)	0.84 (0.15)	0.5–1.5
GAMMA globulins	GGB (%)	21.2 (17.8–26)	22.0–33.5
Gamma-glutamyl transpeptidase	GGT (U/L)	32.7 (9.4)	20–50
Glutamic-pyruvic transaminase	GPT	13.1 (4.7)	11–19
Aspartate aminotransferase	AST (U/L)	95 (84–105)	70–180
Alanine aminotransferase	ALT (U/L)	46 (36–55)	25–75
Total Proteins	TPRO (g/dL)	7.5 (0.6)	6–8.2
Blood urea nitrogen	BUN (mg/dl)	29.8 (9.2)	10–30
White blood cells	WBC (10³/µL)	8.6 (2.2)	7–16.4
Red blood cells	RBC (10^6^/µL)	6.3 (0.9)	5.35–12.1
Hemoglobin	HGB (g/dL)	10.6 (1.1)	8.8–12.9
Hematocrit	HCT (%)	27.2 (2.9)	23.8–35.5
Average corpuscular volume	MCV (fL)	43.3 (4.2)	27.2–47.5
Average hemoglobin content	MCH (pg)	16.9 (1.8)	9.9–18.2
Medium corpuscular hemoglobin concentration	MCHC (g/dL)	38.6 (37.5–40.2)	33.9–38.8
Amplitude of erythrocytes distribution	RDW (%)	18.2 (1.4)	23.3–33.6
Amplitude of hemoglobin distribution	HDW (g/dL)	2.2 (0.2)	2.2–3.1
Platelets	PLT (10³/µL)	402 (147)	140–870
Average platelet volume	MPV (fL)	7.9 (7.2–13.4)	5–8
Total number of neutrophils	NEUT (10³/µL)	3.4 (2.6–4.3)	1.1–3.6
Total number of lymphocytes	LYMPH (10³/µL)	3.6 (2.9–4.5)	4–9.8
Total number of monocytes	MONO (10³/µL)	0.48 (0.35–0.61)	0.2–1.3
Total number of eosinophils	EOS (10³/µL)	0.36 (0.24–0.49)	0– 0.7
Total number of basophils	BAS (10³/µL)	0.07 (0.05–0.09)	0–0.2
Total number of leucocytes	LEU (10³/µL)	5 (3–8)	5–10
Neutrophil-lymphocyte ratio	NLR (10³/µL)	0.9 (0.7–1.2)	*

Unit of measurement: %, percentage; g/dL, grams per decilitre; mg/dL, milligram per decilitre; pg, picogram; fL, femtoliter; U/L, units per litre; 10³/µL, 10^3^ per microlitre; 10^6^/µL, 10^6^ per microliter; * no. laboratory reference range is available given that this measurement is a combination of the total number of neutrophils and the total number of lymphocytes.

**Table 3:** Correlation matrix based on (a) A1–management factor, (b) A2–housing factors, (c) A3–animal-based measures, (d) B–biosecurity, (e) C–risk and alarm system scores as outcomes, and fact1-4 as explicative variables. Data are presented as Spearman’s correlation coefficient for statistically significant variables correlated with score values (*** *p*-Value < 0.0001, ** *p*-Value range (0.0001–0.005) * *p*-Value range (0.005–0.05)).

-	Score A1–Management Factor (a)	A1GB	A2GB	ALB	ALP	GGT	GPT	TPRO	RBC	HCT	MCH	MCHC	HDW	PLT	MPV	EOS	NLR
**Score A1–management factor**	1	-	-	-	-	-	-	-	-	-	-	-	-	-	-	-	-
**A1GB**	−0.36 ***	1	-	-	-	-	-	-	-	-	-	-	-	-	-	-	-
**A2GB**	0.34 ***	−0.60 ***	1	-	-	-	-	-	-	-	-	-	-	-	-	-	-
**ALB**	0.22 *	−0.24 **	−0.03	1	-	-	-	-	-	-	-	-	-	-	-	-	-
**ALP**	0.29 **	0.07	0.06	0.10	1	-	-	-	-	-	-	-	-	-	-	-	-
**GGT**	−0.18 *	0.04	0.07	−0.16	−0.43 ***	1	-	-	-	-	-	-	-	-	-	-	-
**GPT**	−0.48 ***	0.18 *	−0.18 *	0.14	−0.15	0.38 ***	1	-	-	-	-	-	-	-	-	-	-
**TPRO**	−0.22 *	−0.03	−0.10	−0.01	−0.46 ***	0.39 ***	0.09	1	-	-	-	-	-	-	-	-	-
**RBC**	0.18 *	0.04	0.01	0.18 *	0.61 ***	−0.27 **	0.01	−0.43 ***	1	-	-	-	-	-	-	-	-
**HCT**	0.17 *	−0.17 *	−0.04	0.31 **	−0.40 ***	−0.21 *	−0.02	−0.40 ***	0.69 ***	1	-	-	-	-	-	-	-
**MCH**	−0.22 *	−0.04	−0.09	0.07	−0.45 ***	0.17 *	0.13	0.19 *	−0.72 ***	−0.17	1	-	-	-	-	-	-
**MCHC**	−0.20 *	0.33 ***	−0.20 *	0.01	−0.07	0.04	0.24 **	0.16	−0.25 **	−0.42 ***	0.34 ***	1	-	-	-	-	-
**HDW**	0.54 ***	−0.21 *	0.06	0.06	0.22 *	−0.13	−0.37 ***	−0.15	0.21*	0.01	−0.35 ***	0.15	1	-	-	-	-
**PLT**	−0.21 *	0.12	0.14	−0.39 ***	−0.21 *	0.10	−0.20 *	0.25 **	−0.37 ***	−0.41 ***	0.12	−0.08	−0.14	1	-	-	-
**MPV**	0.17 *	0.01	−0.03	0.14	0.07	−0.15	0.01	0.01	−0.05	−0.12	0.22 *	0.64 ***	0.12	−0.27 **	1	-	-
**EOS**	−0.23 *	−0.01	−0.12	0.20	0.02	0.10	0.41 ***	0.11	0.14	0.15	0.01	0.03	−0.16	−0.45 ***	0.17 *	1	-
**NLR**	−0.19 *	−0.01	0.10	−0.31 ***	−0.31 ***	−0.25 **	−0.05	0.32 ***	−0.33 ***	−0.26 **	0.23 **	0.13	−0.22*	0.31 **	0.05	0.06	1
-	**Score** **A2–housing factors (b)**	**A2GB**	**ALP**	**GGT**	**GPT**	**WBC**	**RBC**	**MCV**	**MCH**	**HDW**	**MPV**	**LYMPH**	**NLR**	-	-	-	-
**Score A2–housing factors**	1	-	-	-	-	-	-	-	-	-	-	-	-	-	-	-	-
**A2GB**	0.17 *	1	-	-	-	-	-	-	-	-	-	-	-	-	-	-	-
**ALP**	0.22 *	0.06	1	-	-	-	-	-	-	-	-	-	-	-	-	-	-
**GGT**	−0.20 *	0.07	−0.43 *	1	-	-	-	-	-	-	-	-	-	-	-	-	-
**GPT**	−0.30 **	−0.17 *	−0.18 *	0.40 *	1	-	-	-	-	-	-	-	-	-	-	-	-
**WBC**	0.21 *	0.04	0.39 *	−0.29 *	−0.18 *	1	-	-	-	-	-	-	-	-	-	-	-
**RBC**	0.23 *	0.01	0.61 *	−0.27 *	−0.01	0.49 *	1	-	-	-	-	-	-	-	-	-	-
**MCV**	−0.25 *	0.02	−0.42 *	0.15	0.02	−0.43 *	−0.61 *	1	-	-	-	-	-	-	-	-	-
**MCH**	−0.25 *	−0.09	−0.45 *	0.17 *	0.14	−0.37 *	−0.72 *	0.85 *	1	-	-	-	-	-	-	-	-
**HDW**	0.26 *	0.06	0.22 *	−0.13	−0.37 *	0.13	0.2087 *	−0.29 *	−0.35 *	1	-	-	-	-	-	-	-
**MPV**	0.19 *	−0.03	0.07	−0.15	−0.02	0.13	−0.05	−0.11	0.22 *	0.12	1	-	-	-	-	-	-
**LYMPH**	0.30 **	−0.06	0.58 *	−0.32 *	−0.048	0.65 *	0.57 *	−0.46 *	−0.42 *	0.17 *	0.05	1	-	-	-	-	-
**NLR**	−0.29 **	0.10	−0.31 ***	−0.31 ***	−0.25 **	0.14 *	−0.33 ***	0.18 *	0.23 **	−0.22 *	−0.05	−0.74 ***	1	-	-	-	-
-	**Score** **A3–Animal-based Measures (c)**	**A1GB**	**A2GB**	**BGB**	**GGB**	**MCHC**	**MPV**	**BAS**	-	-	-	-	-	-	-	-	-
**Score A3–animal-based measures**	1	-	-	-	-	-	-	-	-	-	-	-	-	-	-	-	-
**A1GB**	−0.52 ***	1	-	-	-	-	-	-	-	-	-	-	-	-	-	-	-
**A2GB**	0.37 ***	−0.65 ***	1	-	-	-	-	-	-	-	-	-	-	-	-	-	-
**BGB**	−0.32 ***	0.11	−0.01	1	-	-	-	-	-	-	-	-	-	-	-	-	-
**GGB**	0.25 *	−0.03	−0.04	−0.13	1	-	-	-	-	-	-	-	-	-	-	-	-
**MCHC**	0.31 *	0.33 *	−0.20 *	−0.04	0.11	1	-	-	-	-	-	-	-	-	-	-	-
**MPV**	0.27 *	0.01	−0.03	−0.03	0.02	0.64 ***	1	-	-	-	-	-	-	-	-	-	-
**BAS**	0.34 **	0.15	−0.02	0.08	−0.09	0.05	0.24 **	1	-	-	-	-	-	-	-	-	-
-	**Score** **B–Biosecurity (d)**	**A1GB**	**A2GB**	**BGB**	**GPT**	**TPRO**	**BUN**	**HCT**	**MCHC**	**HDW**	**EOS**	-	-	-	-	-	-
**Score B–biosecurity**	1	-	-	-	-	-	-	-	-	-	-	-	-	-	-	-	-
**A1GB**	−0.71 ***	1	-	-	-	-	-	-	-	-	-	-	-	-	-	-	-
**A2GB**	0.72 ***	−0.60 ***	1	-	-	-	-	-	-	-	-	-	-	-	-	-	-
**BGB**	−0.19 *	0.11	−0.01	1	-	-	-	-	-	-	-	-	-	-	-	-	-
**GPT**	−0.51 ***	0.18 *	−0.18 *	0.01	1	-	-	-	-	-	-	-	-		-	-	-
**TPRO**	−0.27 **	−0.03	−0.10	0.04	0.09	1	-	-	-	-	-	-	-	-	-	-	-
**BUN**	−0.22 *	−0.13	0.01	−0.14	0.24 **	0.29 **	1	-	-	-	-	-	-	-	-	-	-
**HCT**	0.19 *	−0.17 *	−0.03	−0.11	−0.02	−0.40 ***	−0.14	1	-	-	-	-	-	-	-	-	-
**MCHC**	−0.26 **	0.33 ***	−0.21 *	−0.09	0.24 **	0.16	−0.12	−0.41 ***	1	-	-	-	-	-	-	-	-
**HDW**	0.35 ***	−0.21 *	0.06	−0.15	−0.37 ***	−0.15	0.04	0.01	−0.15	1	-	-	-	-	-	-	-
**EOS**	−0.25 **	−0.01	−0.12	−0.17 *	0.41 ***	0.11	0.21 *	0.15	0.03	−0.16	1	-	-	-	-	-	
-	**C–risk and Alarm** **System (e)**	**A2GB**	**BUN**	**MCHC**	**HDW**	**PLT**	**BAS**	**NLR**	-	-	-	-	-	-	-	-	-
**C–risk and alarm system**	1	-	-	-	-	-	-	-	-	-	-	-	-	-	-	-	-
**A2GB**	0.23 *	1	-	-	-	-	-	-	-	-	-	-	-	-	-	-	-
**BUN**	0.66 ***	0.01	1	-	-	-	-	-	-	-	-	-	-	-	-	-	-
**MCHC**	−0.37 **	−0.21 *	−0.12	1	-	-	-	-	-	-	-	-	-	-	-	-	-
**HDW**	0.40 **	0.06	0.04	−0.15	1	-	-	-	-	-	-	-	-	-	-	-	-
**PLT**	−0.20 *	0.14	−0.14	0.08	−0.13	1	-	-	-	-	-	-	-	-	-	-	-
**BAS**	0.23 *	−0.02	−0.04	0.05	0.18 *	0.22 *	1	-	-	-	-	-	-	-	-	-	-
**NLR**	−0.22 **	0.10	0.02	0.13	−0.22 *	0.31 **	−0.12	1	-	-	-	-	-	-	-	-	-

## Data Availability

The data presented in this study are available on request from the corresponding author.

## Supplementary Materials

The following are available online at https://www.mdpi.com/2076-2615/11/3/854/s1, Table S1. Structure of welfare protocol, Table S2. Minimum number of animals to be observed for animal-based measures (ABMs) evaluation, Table S3. List of laboratory test parameters analyzed to evaluate the correspondence with animal welfare status observed with AWB-EF, by category (metabolic profile, electrophoresis, blood count).

## References

[1] Brambell F.W.R. (1965). Report of the Technical Committee to Enquire into the Welfare of Animals Kept under Intensive Livestock Husbandry Systems.

[2] Lawrence A.B., Stott A.W. Animal Welfare and profitable farming: Getting the best of both worlds. Proceedings of the 3rd Boehringer Ingelheim Expert Forum on Farm Animal Well-Being.

[3] Fraser D. (1995). Science, Values and animal welfare: Exploring the ‘inextricable connection’. Anim. Welf..

[4] Devitt C., Hanlon A., More S.J., Kelly P.C., Blake M. (2018). Challenges and Solutions to Supporting Farm Animal Welfare in Ireland: Responding to the Human Element. Technical Report in Veterinary Medicine Research Collection. https://www.readkong.com/page/challenges-and-solutions-to-supporting-farm-animal-welfare-4543066.

[5] World Organization for Animal Health (OIE) (2016). Guidelines on Disaster Management and Risk Reduction in Relation to Animal Health and Welfare and Veterinary Public Health. https://www.oie.int/fileadmin/Home/eng/Animal_Welfare/docs/pdf/Others/Disastermanagement-ANG.pdf.

[6] Broom D.M., Johnson K.G. (1993). Stress and Animal Welfare.

[7] More S.J., Hanlon A., Marchewka J., Boyle L. (2017). Private animal health and welfare standards in quality assurance programmes: A review and proposed framework for critical evaluation. Vet. Rec..

[8] Amadori M., Zanotti C. (2016). Immunoprophylaxis in intensive farming systems: The way forward. Vet. Immunol. Immunopathol..

[9] Razzuoli E., Zanotti C., Amadori E. (2016). Modulation of the Interferon Response by Environmental, Noninfectious Stressors. The Innate Immune Response to Noninfectious Stressors; Human and Animal Models.

[10] De Pasillé A.M., Rushen J. Effects of spatial restriction and behavioural deprivation on open field responses, growth and adrenocortical reactivity of calves. Proceedings of the 29th International Congress ISAE.

[11] Jensen M.B. (1999). Effects of confinement on rebounds of locomotor behaviour of calves and heifers, and the spatial preferences of calves. Appl. Anim. Behav. Sci..

[12] Bouissou M.F., Boissy A., Le Neindre P., Veissier I., Keeling L., Gonyou H. (2001). The social behaviour of cattle. Social Behaviour in Farm Animals.

[13] Rousing T., Wemelsfelder F. (2006). Qualitative assessment of social behaviour of dairy cows housed in loose housing systems. Appl. Anim. Behav. Sci..

[14] Wemelsfelder F., De Rosa G., Napolitano F. (2006). Qualitative Indicators for the On-Farm Monitoring of Cattle Welfare. EU-Project Welfare Quality. http://www.welfarequality.net.

[15] Rushen J., De Passillé A.M., Von Keyserlingk M.A.G., Weary D.M. (2008). The Welfare of Cattle.

[16] Napolitano F., Knierim U., Grass F., De Rosa G. (2009). Positive indicators of cattle welfare and their applicability to on-farm protocols. Ital. J. Anim. Sci..

[17] Capdeville J., Veissier I. (2001). A method of assessing welfare in loose housed dairy cows at farm level, focusing on animal observations. Acta Agric. Scand. Sect. A Anim. Sci..

[18] Farm Animal Welfare Council (FAWC) (2009). Report on Farm Animal Welfare in Great Britain: Past, Present and Future.

[19] Knierim U., Carter C.S., Fraser D., Gartner K., Lutgendorf S.K., Mineka S., Panksepp J., Sachser N., Broom D.M. (2001). Group report: Good welfare. Improving quality of life. Welfare in Animals Including Humans.

[20] Botreau R., Veisser I., Butterworth A., Bracke M.B.M., Keeling L.J. (2007). Definition of criteria for overall assessment of animal welfare. Anim. Welf..

[21] Webster A.J.F. (2009). The virtuous bicycle: A delivery vehicle for improved farm animal welfare. Anim. Welf..

[22] Peli A., Pietra M., Giacometti F., Mazzi A., Sacco G., Serraino A., Scagliarini L. (2016). Survey on Animal Welfare in Nine Hundred and Forty Three Italian Dairy Farms. Ital. J. Food Saf..

[23] EFSA (2012). Scientific opinion of the panel on Animal Health and Welfare on the use of animal-based measures to assess welfare of dairy cows. EFSA J..

[24] EFSA (2012). Guidance on Risk Assessment for animal Welfare. EFSA J..

[25] Bertocchi L., Fusi F., Angelucci A., Bolzoni L., Pongolini S., Strano R.M., Ginestreti J., Riuzzi G., Moroni P., Lorenzi V. (2018). Characterization of hazards, welfare promoters and animal-based measures for the welfare assessment of dairy cows: Elicitation of expert opinion. Prev. Vet. Med..

[26] (2019). Vetinfo, Statistiche BDN. https://www.vetinfo.it/j6_statistiche/#/report-list/2.

[27] Corian C.O., Miresan V., Corian A., Raducucu C., Andronie L., Marchis Z., Terhes S., Muntean M.V. (2017). Biochemical and Haematological Blood Parameters at Different Stages of Lactation in Cows. Bulletin UASVM. Anim. Sci. Biotechnol..

[28] Radkowska I., Herbut E. (2014). Hematological and biochemical blood parameters in dairy cows depending on the management system. Anim. Sci. Pap. Rep..

[29] Simonov M., Vlizlo V. (2015). Some blood markers of the functional state of liver in dairy cows with clinical ketosis. Bulg. J. Vet. Med..

[30] Italian Ministry of Health (1998). Attuazione Della Direttiva 97/2/CE Relativa Alle Norme Minime Per La Protezione Dei Vitelli. Legislative Decree No. 331, Gazzetta Ufficiale No. 224. https://www.gazzettaufficiale.it/eli/id/1998/09/25/098G0384/sg.

[31] ISTAT (2017). Annuario Statistico Italiano. https://www.istat.it/it/archivio/213021.

[32] Regione Sardegna (2016). Riordino Del Sistema Delle Autonomie Locali Della Sardegna. Regional Low No. 2. https://www.regione.sardegna.it/j/v/80?s=300929&v=2&c=13906&t=1#:~:text=2,-Riordinodelsistema&text=Lapresenteleggedisciplinal,StatutospecialeperlaSardegna.

[33] Agris Relazione Previsionale e Programmatica di Accompagnamento al Bilancio di Previsione Anni 2014/2016, Bilancio di Previsione Per L’anno 2014 e Bilancio Pluriennale Per Gli Anni 2014–16. http://sardegnaagricoltura.it/.

[34] Tothova C., Nagy O., Kovac G. (2016). Serum proteins and their diagnostic utility in veterinary medicine: A review. Vet. Med..

[35] EFSA (2009). Scientific opinion of the panel on Animal Health and Welfare on a request from the Commission on the risk assessment of the impact of housing, nutrition and feeding, management and genetic selection on behaviour, fear and pain problems in dairy cows. EFSA J..

[36] EFSA (2009). Scientific opinion of the panel on Animal Health and Welfare on a request from the Commission on the risk assessment of the impact of housing, nutrition and feeding, management and genetic selection on metabolic and reproductive problems in dairy cows. EFSA J..

[37] EFSA (2009). EFSA Scientific opinion of the panel on Animal Health and Welfare on a request from the Commission on the risk assessment of the impact of housing, nutrition and feeding, management and genetic selection on udder problems in dairy cows. EFSA J..

[38] EFSA (2009). Scientific opinion of the panel on Animal Health and Welfare on a request from the Commission on the risk assessment of the impact of housing, nutrition and feeding, management and genetic selection on leg and locomotion problems in dairy cows. EFSA J..

[39] EFSA (2009). Scientific report of EFSA prepared by the Animal Health and Animal Welfare Unit on the effects of farming systems on dairy cow welfare and disease. EFSA J..

[40] EFSA (2014). Guidance on expert knowledge elicitation in food and feed safety risk assessment. EFSA J..

[41] EFSA (2015). Scientific opinion on the assessment of dairy cow welfare in small-scale farming systems. EFSA J..

[42] Anderson J.C., Gerbing D.W. (1984). The effect of sampling error on convergence, improper solution, and goodness-of-fit indices for maximum likelihood confirmatory factor analysis. Psychometrika.

[43] MacCallum R.C., Widaman K.F., Zhang S., Hong S. (1999). Sample size in factor analysis. Psychol. Methods.

[44] Jolliffe J.T., Cadima J. (2016). Principal component analysis: A review and recent developments. R. Soc..

[45] Tyler D.E. (1981). Asymptotic inference for eigenvectors. Ann. Stat..

[46] Rousseeuw P.J., Hubert M. (2011). Robust statistics for outlier detection. WIREs Data Min. Knowl. Discov..

[47] Edwards A.L. (1976). An Introduction to Linear Regression and Correlation.

[48] Bonizzi L., Amadori M., Melegari M., Ponti W., Ceccarelli A., Bolzani E. (1989). Characterization of some parameters of non-specific immunity in dairy cattle (I). Zent. Vet. B..

[49] Razzuoli E., Olzi E., Calà P., Cafazzo S., Magnani D., Vitali A., Lacetera N., Archetti L., Lazzara F., Ferrari A. (2016). Innate immune responses of young bulls to a novel environment. Vet. Imm. Immunopathol..

[50] Amadori M., Archetti L., Frasnelli M., Bagni M., Olzi E., Caronna G., Lanteri M. (1997). An immunological approach to the evaluation of welfare in Holstein Frisian cattle. Zent. Vet. B..

[51] Hagawane S., Shinde S.B., Rajguru D.N. (2009). Haematological and Blood Biochemical Profile in Lactating Buffaloes in and around Parbhani city. Vet. World.

[52] Sharma V., Sridhar S. (2007). Evaluation of Some Liver Function Tests in Clinical Cases of Hepatic Insufficiency in Buffaloes. Ital. J. Anim. Sci..

[53] Aguirre E.L., Quezada M., Uchuari M., Mamani G. (2018). ALP-AST/GOT-ALT/GPT-Bilirubin in Serum from Bos Taurus Cows in the Postpartum Period and Maintained by Grazing in the Humid Tropic Region. ARC J. Anim. Vet. Sci..

[54] Mudron P., Rehage J., Qualmann K., Sallmann H.P., Scholz H. (1999). A study of lipid peroxidation and vitamin E in dairy cows with hepatic insufficiency. J. Vet. Med. A..

[55] Broom D.M. (1988). The scientific assessment of animal welfare. Appl. Anim. Behav. Sci..

[56] Fraser D. (2008). Understanding animal welfare. Acta Vet. Scand..

[57] Grunwaldt E.G., Guevara J.C., Estevez O.R., Vicente A., Rousselle H., Alcunten N., Aguerregaray D., Stasi C.R. (2005). Biochemical and haematological measurements in beef cattle in Mendoza plain rangelands (Argentina). Trop. Anim. Health Prod..

[58] Vatn S., Framstad T., Torsteinbø W.O. (2000). Hematologic evaluation of normal and anemic lambs with the Technicon H* 1 using EDTA or heparin as anticoagulants. Vet. Clin. Pathol..

[59] Trevisi E., Amadori M., Cogrossi S., Razzuoli E., Bertoni G. (2012). Metabolic stress and inflammatory response in high-yielding, periparturient dairy cows. Res. Vet. Sci..

[60] Broom D.M. (1986). Indicators of poor welfare. Br. Vet. J..

[61] Bracke M.B.M., Edwards S.A., Engel B., Buist W.G., Algers B. (2008). Expert opinions ‘validation’ of risk assessment applied to calf welfare. Acta Vet. Scand..

[62] Mullan S., Edwards S.A., Butterworth A., Whay H.R., Main D.C.J. (2011). Inter-observer reliability testing of pig welfare outcome measures proposed for inclusion within farm assurance schemes. Vet. J..

[63] Phythian C.J., Michalopoulou E., Jones P.H., Winter A.C., Clarkson M.J., Stubbings L.A., Grove W.D., Cripps P.J., Duncan J.S. (2011). Validating indicators of sheep welfare through a consensus of expert opinion. Animal.

[64] Sørensen J.T., Fraser D. (2010). On-farm welfare assessment for regulatory purposes: Issues and possible solutions. Livest. Sci..

[65] Chau Dang Van Q., Knapp E., Hornick J.L., Dufrasne I. (2020). Influence of days in milk and parity on milk and blood fatty acid concentrations, blood metabolites and hormones in early lactation Holstein cows. Animals.

