# Supplementary material for: Welfare Assessment: Correspondence Analysis of Welfare Score and Hematological and Biochemical Profiles of Dairy Cows in Sardinia, Italy
Source: Animals (Basel). 2021 Mar 17;11(3):854. doi: 10.3390/ani11030854 (PMC8002757; doi:10.3390/ani11030854)
Supplement: Supplementary file 1 [file animals-11-00854-s001.pdf]

Table S1. Structure of welfare protocol.

Item	Section A1: Management factors
1	Number of stockpeople (ALL)
2	Experience and training of stockpersons (ALL)
3	Animal grouping strategy (LC, DC, HF)
4	Inspections of animals (ALL)
5	Type of handling (LC)
6	Milking parlor access and exit (LC)
7	Feeding strategy (LC, DC, HF)
8	Use of concentrated feeds (daily dose) (LC)
9	Water provision (ALL)
10	Cleanliness of water points (LC)
11	Cleanliness of water points (DC)
12	Cleanliness of water points (HF)
13	Cleanliness of floor in walking areas (LC)
14	Cleanliness of floor in walking areas (DC)
15	Cleanliness of floor in walking areas (HF)
16	Bedding material management (LC)
17	Bedding material management (DC)
18	Use and management of calving pens (DC)
19	Bedding material management (HF)
20	Foot inspection and foot bathing (LC, DC)
21	Hygiene (milking parlor or robot) (LC)
22	Milking routine (LC)
	Section A2: Housing factors
1	Housing of animals older than 6 months (LC, DC, HF)
2	Space availability in lying area (LC)
3	Space availability in lying area 8 (DC)
4	Calving pen presence and size (DC)
5	Space availability in lying area (HF)
6	Design of lying area (LC)
7	Type of bedding material (LC)
8	Type of bedding material (DC)
9	Type of bedding material (HF)
10	Type of floor in walking areas (LC)
11	Type of floor in walking areas (DC)
12	Type of floor in walking areas (HF)
13	Available space at feed bunk (LC)
14	Available space at feed bunk (DC)
15	Available space at feed bunk (HF)
16	Feeding place dimension and accessibility (LC)
17	Function and number of water points (LC)
18	Function and number of water points (DC9)
19	Function and number of water points (HF)
20	Bedding material for new-born calves in single pens (CV)
21	Space availability for calves up to 8 weeks (CV)
22	Possibility for calves to see and touch each other (CV)
23	Space availability for calves in group pen (CV)
24	Facilities for sick animals (ALL)

25	Waiting room and milking parlor design (LC)
26	Milking machine or robot maintenance (LC)
27	Temperature, humidity, and ventilation (ALL)
28	Gas (NH3, H2S, CO2) concentration (ALL)
29	Use of artificial lighting (ALL)
	Section A3: Animal-based measures
1	Lameness (LC, DC)
2	Annual mortality rate (CV)
3	Annual mortality rate (LC DC)
4	Antibiotic treatments for mastitis (LC)
5	Body condition score (LC)
6	Integument alterations (LC)
7	Body condition score (DC)
8	Integument alterations (DC)
9	Avoidance distance test (LC)
10	Mutilations (ALL)
11	Avoidance distance test (DC)
12	Integument alteration (HF)
13	Body condition score (HF)
14	Milk somatic cell count (LC)
15	Avoidance distance test (HF)
16	Cleanliness of flank, leg, udder (LC)
17	Cleanliness of flank, leg, udder (DC)
18	Cleanliness of flank, leg, udder (HF)
	Section B: Biosecurity
1	Biosecurity procedures in the fight against rodents and insects
2	Presence of other animal species within the farm
3	General precautions for entry of strangers
4	Management of the entry of routine visitors
5	Disinfection of vehicles at the entrance to the farm
6	Possibility of contact between foreign vehicles and bred animals
7	Collection of dead animal carcasses
8	Loading of live animals (e.g., for sale)
9	Buying and/or moving animals out of the farm
10	Quarantine
11	Control and prevention of mastitis
12	Control and prevention of major infectious diseases
13	IBR health condition
14	Paratuberculosis health condition
15	Control and analysis of water sources
	Section C: Risks and alarm system
1	Origin of drinking water
2	Noise
3	Ventilation system alarm
4	Fire alarm
5	Electricity generator

All = all animal groups; DC = dry cows; HF = heifers; LC = lactating cows.

Table S2. Minimum number of animals to be observed for animal-based measures (ABMs) evaluation.

Population (N)	Sample (n)
≤ 30	All animals
31–99	30–39
100–199	40–50
200–299	51–55
300–549	55–59
550–1000	60–63
1001–3000	63–65

Table S3. List of laboratory test parameters analyzed to evaluate the correspondence with animal welfare status observed with AWB-EF, by category (metabolic profile, electrophoresis, blood count).

Acronym	Parameter	Exam Type
WBC	White blood cells	Blood count
RBC	Red blood cells	Blood count
HGB	Hemoglobin	Blood count
HCT	Hematocrit	Blood count
MCV	Average corpuscular volume	Blood count
MCH	Average hemoglobin content	Blood count
MCHC	Medium corpuscular hemoglobin concentration	Blood count
RDW	Amplitude of erythrocytes distribution	Blood count
HDW	Amplitude of hemoglobin distribution	Blood count
PLT	Platelets	Blood count
MPV	Average platelet volume	Blood count
NEUT%	Percentage of Neutrophils	Blood count
LYMPH%	Percentage of Lymphocytes	Blood count
MONO%	Percentage of Monocytes	Blood count
EOS%	Percentage of Eosinophils	Blood count
BASO%	Percentage of Basophils	Blood count
NEUT	Total number of Neutrophils	Blood count
LYMPH	Total number of Lymphocytes	Blood count
MONO	Total number of Monocytes	Blood count
EOS	Total number of Eosinophils	Blood count
BASO	Total number of Basophils	Blood count
A1GB	ALFA1 globulins	Electrophoresis
A2GB	ALFA2 globulins	Electrophoresis
BGB	BETA globulins	Electrophoresis
GGB	GAMMA globulins	Electrophoresis
ALB	Albumin	Metabolic profile
ALP	ALP	Metabolic profile
TB	Total Bilirubin	Metabolic profile
CREA	Creatinine	Metabolic profile
GGT	GGT	Metabolic profile
GOT	GOT/ASAT	Metabolic profile
GPT	GPT/ALAT	Metabolic profile
TPRO	Total Proteins	Metabolic profile
BUN	Urea	Metabolic profile
